# Willingness Towards and Associated Factors in Receiving COVID-19 Vaccination During and After the Pandemic

**DOI:** 10.3390/vaccines14020162

**Published:** 2026-02-09

**Authors:** Paul Shing-fong Chan, Zixin Wang

**Affiliations:** 1 Department of Psychology, The Education University of Hong Kong, Hong Kong 999077, China; 2 Jockey Club School of Public Health and Primary Care, The Chinese University of Hong Kong, Hong Kong 999077, China

## 1. Introduction

As Guest Editors of the Special Issue “Trust, Willingness, and Associated Factors towards COVID-19 Vaccine Uptake” [1], we are pleased to reflect on the contributions of seven papers published in this Special Issue [2,3,4,5,6,7,8]. These studies, conducted across diverse global contexts, explore the multifaceted influences on vaccine acceptance, from individual beliefs to socio-cultural barriers. On 5 May 2023, the World Health Organization (WHO) declared that COVID-19 was no longer a public health emergency of international concern (PHEIC). Notably, in this Special Issue, five papers were based on research conducted before 5 May 2023, one spanned before and after 5 May 2023, and one was executed entirely after the declaration. This Special Issue addresses critical themes such as parental decision-making, health worker roles, and vaccine hesitancy in chronic disease patients.

In this Editorial, we present the current global context and updated WHO Strategic Advisory Group on Immunization (SAGE)’s recommendations, a quick systematic review of studies conducted after 5 May 2023, an overview of the seven papers published in this Special Issue, and priorities for future interventions to address barriers to COVID-19 vaccination uptake.

## 2. Current Global Context and Updated SAGE Recommendations for COVID-19 Vaccination

### 2.1. Recent Data on COVID-19 Cases and Deaths

According to the WHO, from 24 to 30 November 2025, a total of 55,318 samples from 81 countries were tested for SARS-CoV-2, the virus that causes COVID-19 [9]. The WHO compiled these figures from an international system of monitoring and surveillance facilities. Among the samples, 2557 (4.6%) returned positive results [9]. Overall, global SARS-CoV-2 activity was stable during this timeframe. Additionally, the number of COVID-19 deaths reported to the WHO was 885 in the 28 days to 30 November 2025, including the United States (*n* = 652), Sweden (*n* = 60), Brazil (*n* = 54), Greece (*n* = 29), Portugal (*n* = 19), and Czechia (*n* =17) [9].

### 2.2. Updated SAGE Recommendations for COVID-19 Vaccination

The WHO SAGE recommendations for COVID-19 vaccination prioritize initiating and maintaining protection, especially for vulnerable groups. For those who have never been vaccinated, all adults should receive one dose to build baseline immunity, while children and adolescents with comorbidities are advised to receive one dose to prevent severe outcomes. Health workers with patient contact should receive one dose due to exposure risks. Pregnant individuals are encouraged to take one dose during pregnancy for safe maternal and fetal protection, and immunocompromised people need 2–3 doses, with at least 2 to ensure an effective response [10].

For those with at least one prior dose, revaccination sustains immunity, with timing based on risk. High-risk older adults (over 75–80, adjustable by country) should revaccinate 6–12 months after their last dose. Those over 50–60 with comorbidities or immunocompromised conditions should follow the same interval. Adults over 50–60 without comorbidities, or with them, are recommended revaccination after 12 months. Health workers should revaccinate annually due to ongoing exposure, while pregnant people need one dose per pregnancy. In contrast, healthy adults, as well as children and adolescents without comorbidities, do not require routine revaccination, reflecting the lower overall risk in these groups [10].

## 3. Quick Systematic Review on Studies Conducted After Declaring COVID-19 Is No Longer a PHEIC

We conducted a quick systematic review of the willingness towards and factors associated with COVID-19 vaccination in the post-pandemic era (studies conducted after 5 May 2023). A total of 16 studies were identified [11,12,13,14,15,16,17,18,19,20,21,22,23,24,25,26].

Studies were conducted across different countries including Bangladesh [11], Canada [12], Chinese Mainland [13], France [14], Greece [15], Indonesia [16], Italy [17], Japan [18,19], Malta [20], South Korea [21], Thailand [22], United States [23,24], Zambia [25], and multi-coutries [26]. The prevalence of willingness/uptake differed by dose. Regarding primary doses, for instance, willingness ranged from 77 to 99% [12,13,20]. Willingness to take up booster doses was lower, ranging from 17 to 82% [14,18,23]. Positive factors associated with higher willingness included older age [12,13,14,18], higher education [21,23,26], trust in vaccines/doctors/government [13,20,26], perceived susceptibility/efficacy [13,21], physician recommendations [15], and prior vaccination [13,23]. Negative factors included female gender [14,15,23], safety concerns/side effects [11,15,16,17,18,20,26], low perceived risk [12,15,18,26], and misinformation [26]. The aforementioned positive and negative factors echoed those found in the seven papers published in this Special Issue and previous systematic reviews which were conducted during the pandemic [27,28,29].

## 4. An Overview of the Seven Published Articles

The Special Issue features seven studies on COVID-19 vaccine willingness, spanning pre- and post-PHEIC periods. Pre-May 2023 research, including surveys in Hong Kong, Germany, Bangladesh, the UK, and Gaza, emphasized factors like health perceptions, trust in authorities, and health worker influence on uptake among older adults, parents, pregnant women, and general populations. A U.S. study bridging timelines developed a toolkit for professionals, focusing on literacy and trust-building to combat misinformation. Post May research in Greece highlighted hesitancy in chronic disease patients, which was linked to demographics and concerns. Collectively, these studies underscore tailored interventions to address barriers and enhance vaccination through education and support.

### 4.1. Studies Conducted Before 5 May 2023

Liang et al. conducted their study from November 2021 to January 2022 in Hong Kong, China [2]. This study aimed to examine the associations between COVID-19 vaccination, related perceptions, and behavioral intention to receive seasonal influenza vaccination (SIV) among older adults aged 65 years or above. A random telephone survey was conducted among 440 community-dwelling older adults. The key findings revealed that more than half of the participants intended to receive SIV in the next year. After adjusting for background factors, concerns about negative interactions between SIV and COVID-19 vaccination correlated with lower intention, while perceived higher co-infection risk, severe influenza consequences, SIV benefits, cues from doctors/family/friends, and norms of peer uptake were linked to higher intention. The authors suggested that future programs should address perceptions of both vaccinations concurrently.

Purrmann et al.’s study was conducted from December 2021 to January 2022 in Germany [3]. This study aimed to investigate parents’ willingness to vaccinate children aged 5–11 years against COVID-19, determinants, and beliefs influencing decisions through descriptive analysis and bivariate correlations with mental health status, general vaccination attitudes, and perceptions of SARS-CoV-2 policies. A cross-sectional survey included 2401 parents. The results indicated a higher rate of vaccination uptake compared with refusal. Higher acceptance correlated with parents’ own full vaccination status, personal COVID-19 immunizations, and elevated COVID-19 fear, while refusal was linked to perceived campaign pressure, social restrictions, and opposition to protective measures. No strong ties to mental health emerged except general anxiety. The researchers recommended that future campaigns should minimize pressure, build trust, and address pathogen familiarity distinctions.

Fesshaye and colleagues implemented their study from April to August 2022 in Bangladesh [4]. This study aimed to identify facilitators and barriers to COVID-19 vaccine acceptance and promotion among pregnant and lactating women, given their elevated risk of severe complications. Forty in-depth interviews were conducted using a grounded theory approach with 12 pregnant women, 12 lactating women, and 16 health workers from one urban and four rural communities. The key findings highlighted that health workers and religious leaders, along with trust in public health authorities and existing vaccine infrastructure, facilitated vaccine promotion, while changes in eligibility and myths/rumors served as both facilitators and barriers. Promoting maternal immunization is essential to enhance acceptance, protect maternal and infant health, and inform strategies for future vaccines.

Dasgupta et al. executed their research from May 2022 to February 2023 in the United Kingdom [5]. This study aimed to explore perceptions of COVID-19 vaccination during pregnancy, focusing on marginalized populations and those with social or medical complexities, to understand factors influencing uptake. Ninety-six semi-structured in-depth interviews were conducted across the UK’s four nations with 40 pregnant women, 15 partners, 21 healthcare professionals, and 20 policymakers, discussing their experiences with vaccine utilization, delivery, and policy during the pandemic. The key outcomes revealed three themes: historical and social context (e.g., mistrust in pregnancy drugs offset by prior positive vaccine experiences), communication challenges (missing, conflicting, or false information and confusing guidance), and appraisal leading to actions. The recommendations emphasized personalized information delivery, building trust, and leveraging positive past experiences to enhance vaccination perceptions and uptake.

Majer and colleagues undertook their exploration in March 2023 in the Gaza Strip, Palestine [6]. This follow-up study aimed to re-evaluate COVID-19 vaccination levels, hesitancy, exposure to promotion efforts, and associated risk factors among Gazan adults in March 2023, amid war-devastated health infrastructure, building on a 2021 survey showing low uptake. A community-based cross-sectional survey employed multistage stratified sampling, analyzing associations via bivariate and multivariable logistic regression. The key outcomes indicated that vaccination rates increased notably, with hesitancy showing a slight decline. Referrals from health workers were associated with substantially higher odds of vaccination, while receiving vaccine information from them correlated with lower odds of hesitancy. These findings highlight the crucial role of health workers in promoting vaccination and suggest investing in community health workers’ skills to achieve better results.

### 4.2. Conducted Before and After 5 May 2023

Austin et al. conducted their study from spring 2022 (surveys) to June 2023 in the United States [7]. This study aimed to develop an Integrative Model of Sustainable Health Decision-Making and a toolkit to empower U.S. Extension professionals with knowledge and skills for adult immunization education, thereby reducing mistrust and boosting confidence in vaccine promotion. Utilizing an explanatory parallel mixed methods design, data were collected via needs assessment surveys, interviews, workshops, and neuromarketing message testing among Extension professionals, with the toolkit pilot-tested. The key outcomes identified four primary needs: role-tailored training, preserving community trust and credibility, linking with medical experts, and enhancing science media literacy to combat misinformation. Correlations validated the model, centering on science media literacy, motivational interviewing, and neuromarketing to foster communication and sustainable well-being, such as improved vaccine uptake.

### 4.3. Conducted After 5 May 2023

Bouloukaki et al. executed their investigation from October to December 2023 in Crete, Greece [8]. This cross-sectional study aimed to assess the prevalence of hesitancy toward regular COVID-19 booster vaccination and identify influencing factors among patients with asthma or chronic obstructive pulmonary disease (COPD) visiting primary care centers. A total of 264 participants completed questionnaires on socio-demographics, health status, vaccination history, attitudes, and beliefs. Multivariate logistic regression was used to analyze associations. The key outcomes showed more than half of the participants had hesitancy, and it was positively linked to female gender, middle age, lower education, depression, side effect concerns, low vaccine efficacy confidence, and media reliance. Conversely, cardiovascular disease, type 2 diabetes, physician adherence, prior flu vaccination, and >3 COVID-19 doses correlated with reduced hesitancy, informing targeted strategies to enhance uptake.

## 5. Recommendations for Future Interventions

The transition to a post-PHEIC era underscores the need for research on innovative interventions, strategies, and policies to sustain COVID-19 vaccine uptake, particularly amid lower booster willingness and priority groups. Future studies should prioritize randomized controlled trials evaluating multifaceted interventions, such as tailored artificial intelligence-based digital campaigns to address safety concerns and misinformation, which are key barriers. For older adults, who showed higher willingness, research could test community-based strategies like subsidized mobile clinics to improve the uptake. For other priority groups, including pregnant women and those with comorbidities or immunocompromised conditions, community health worker-led or peer-led outreach could mitigate barriers. Longitudinal designs tracking vaccine literacy and behavioral nudges (e.g., reminders via apps) across diverse settings are also recommended.

## 6. Conclusions

This Special Issue illuminates the evolving landscape of COVID-19 vaccine trust and willingness, with seven papers highlighting prevalence rates of 35–90% and key factors like safety concerns, physician recommendations, and socioeconomic barriers across diverse populations in various countries/regions. The contributions underscore that while primary uptake has improved, booster hesitancy persists post PHEIC, signaling risks to routine immunization. These insights emphasize the need for sustained, evidence-based efforts to remove barriers, address disparities, and rebuild trust. By advancing targeted strategies, we can enhance global preparedness for ongoing and future health threats.

## Data Availability

Data are available upon request from the corresponding author.
